# Potential Reduction in Adverse Events and Cost with Novel Anticoagulants among Patients with Acute Limb Ischemia

**DOI:** 10.1155/2022/3786815

**Published:** 2022-06-07

**Authors:** Scott Freeman, Piper Williams, Anna E. Barón, Mary E. Plomondon, Stephen W. Waldo

**Affiliations:** ^1^Department of Medicine, Rocky Mountain Regional VA Medical Center, Denver, CO, USA; ^2^University of Colorado School of Medicine, Aurora, CO, USA; ^3^Department of Biostatistics and Informatics, Colorado School of Public Health, University of Colorado Anschutz Medical Campus, Aurora, CO, USA; ^4^VHA Office of Quality and Patient Safety, Washington DC, USA

## Abstract

**Background:**

Acute limb ischemia (ALI) is associated with significant morbidity and mortality. Novel anticoagulants reduce adverse events among patients with peripheral artery disease, though the potential effect of these therapies is unclear in patients with ALI. The present study thus sought to evaluate the potential clinical benefit of universal application of novel anticoagulants to a high-risk population of patients with ALI.

**Methods:**

In this retrospective cohort study, we identified patients diagnosed with ALI in the Veterans Affairs Healthcare System between 2015 and 2016. We then calculated the incidence of adverse cardiovascular events (death/stroke/myocardial infarction/amputation/repeat intervention) as if they were treated with rivaroxaban using published data. Further, we calculated the cost to treat a Veteran diagnosed with one of these outcomes, and the potential savings had patients been universally treated with novel anticoagulants.

**Results:**

We identified 286 patients that presented with lower extremity ALI and were not treated with anticoagulation. Potential treatment of these patients with rivaroxaban resulted in significantly fewer adverse events, with an 11.9% reduction in cases at 21 months (95% CI: 5.5-17.8%) and a 13.4% reduction in cases at 47 months (95% CI: 5.6-20.5%). This corresponded to significant decreases in healthcare spending for patients with ALI who were treated with rivaroxaban.

**Conclusions:**

Among patients with ALI, treatment with rivaroxaban could result in a significant reduction in adverse cardiovascular events. The reduction in events would in turn lead to significant decreases in healthcare spending for this population.

## 1. Background

Peripheral artery disease (PAD) is associated with significant morbidity and mortality, afflicting approximately 8–12 million Americans [1]. Acute limb ischemia (ALI) remains the most catastrophic complication of PAD, most commonly resulting from rupture of an existing atherosclerotic plaque and instigated by thromboembolism and trauma [2]. ALI occurs relatively infrequently with a reported incidence of 1.5 per 10,000 person-years [3]. However, patients suffering from this condition have a remarkably poor prognosis with a high proportion requiring amputation or suffering death within one year of their index presentation [4]. Similarly, the costs related to the management after ALI poses a significant burden both to the patients and the healthcare systems that care for them [5].

The development of therapies to improve outcomes for patients with PAD and ALI has thus been an intense area of research. Conventional antithrombotic therapies have been evaluated in this population, with the Warfarin Antiplatelet Vascular Evaluation (WAVE) Trial failing to show a reduction in adverse events for a broad population of patients with PAD prescribed warfarin in addition to aspirin [6]. Novel antithrombotic therapies, however, have demonstrated improved outcomes with the Vascular Outcomes Study of ASA along with Rivaroxaban in Endovascular or Surgical Limb Revascularization for PAD (VOYAGER PAD) and the Cardiovascular Outcomes for People Using Anticoagulation Strategies (COMPASS) both demonstrating benefits with the use of rivaroxaban [7, 8]. The utilization of these therapies in patients with the most acute manifestations of peripheral artery disease has been underwhelming thus far, including those with ALI.

With this in mind, the present study sought to evaluate the potential clinical benefit of universal application of novel anticoagulants to a high-risk population of patients with ALI. Further analyses sought to evaluate the potential cost effectiveness of this therapy, using reductions in clinical outcomes observed in prior trials evaluating the broader population of patients with PAD.

## 2. Methods

### 2.1. Population

All patients presenting to Veterans Health Administration Hospitals with a new diagnosis of lower extremity ALI and discharged from October 2015 to September 2016 were included in the analysis. For the purpose of this project, hospital admission for acute limb ischemia was first determined using administrative billing codes (ICD-10 code I70, I72-I74, I75.021, I75.022, I77.1, I77.4, T82, T85, and T87). Thereafter, the cases were reviewed by experienced clinicians to confirm that the patient suffered from an acute occlusion of a lower extremity artery resulting in symptoms compatible with acute limb ischemia (SWW). The study was reviewed and approved by the Colorado Multiple Institution Review Board, with a waiver of informed consent.

### 2.2. Measurements

Clinical characteristics of the patient population were derived from the electronic health record. The primary medical therapy for included patients was ascertained from pharmacy data linked to the same electronic medical record. The initial treatment modality was determined based on procedural codes for endovascular (CPT: 37220/37221/37222/37223/37224/37225/37226/37227/37228/37229/37230/37231/37232/37233/37234/37235) and surgical (CPT: 35500/35521/35533/35537/35538/35539/35540/35541/35546/35548/35549/35551/35556/35558/35563/35565/35566/35583/35585/35587/35621/35623/35646/35647/35651/35654/35656/35661/35663/35665/35666/35671/35681/35682/35700 or 35683/35686/35571 in combination with one of the following: 35556/35566/35571/35583/35585/35587/35623/35656/35666/35671) revascularization or primary amputation (CPT: 27880/27881/27882/27884/27886/27590/27591/27592/27594/27596).

### 2.3. Outcomes

The primary outcome was the time to major adverse cardiovascular limb event, comprising cardiovascular death, readmission for stroke, readmission for myocardial infarction, major amputation, or repeat peripheral revascularization consistent with prior publications [7, 8]. A secondary outcome of BARC3a bleeding events, defined as a hemoglobin decline > 3 g/dL or transfusion, was also ascertained within 72 hours of the hospitalization. Outcomes were obtained from the VA electronic health record as well as community care data sources. Mortality was ascertained from the Veterans Health Administration Vital Status File. The cause of death, cardiovascular or noncardiovascular, was determined via chart review from an experienced clinician (SWW). The time to first event was calculated for each patient and those that did not experience the event were censored at 47 months (1,430 days) following their index discharge date, consistent with the maximum follow-up time in the COMPASS trial [7]. The average total cost associated with the five events of the composite outcome was estimated using VA and community care payment data. This total cost consisted of both inpatient and outpatient costs corresponding to the first event. Cardiovascular death was assumed to cost $0.

### 2.4. Analysis

Comparisons were made between those identified with acute limb ischemia, and the cohort was previously analyzed in the COMPASS trial [7, 8]. To do so, Welch's two-sample *t*-test was used to compare continuous variables and Chi-square tests were used to evaluate categorical variables. The number of potential cases avoided was estimated using a cause-specific competing risks Cox proportional hazards model. Non-cardiovascular death was treated as a competing risk in the model. We first calculated the observed cumulative incidence and cumulative hazard for the five-event composite outcome in the ALI veteran cohort. The observed cumulative hazard from the ALI veteran cohort was then multiplied by the hazard ratio, its lower confidence interval (CI) limit, and its upper confidence interval limit reported in the COMPASS trial subgroup analysis (HR = 0.69, 95% CI: 0.56-0.85) to estimate the projected cumulative hazard and 95% CI for the veteran cohort [8]. Then, we transformed the projected cumulative hazard to the projected cumulative incidence of the composite outcome. Finally, we subtracted the projected cumulative incidence from the observed cumulative incidence to get the absolute risk reduction. That percentage was then multiplied by the sample size of the ALI veteran cohort to get the number of potential cases averted. The number of cases averted was calculated at 21 months (630 days) and 47 months (1,430 days) which are the median and maximum follow-up times from the COMPASS trial, respectively. We then multiplied the projected cases averted by the average total cost of the events (and the 95% CI) to estimate the potential cost savings of treating the ALI veteran cohort with rivaroxaban. All analyses were conducted in R version 4.0.2.2. A *p* value < 0.05 was considered statistically significant.

## 3. Results

### 3.1. Population

Of the 329 Veterans diagnosed with ALI in Fiscal Year 2016, 43 were prescribed rivaroxaban at least once following their index admission date and were excluded, leaving a total of 286 veterans for the final analysis. Compared to the COMPASS PAD cohort, Veterans in the ALI cohort were more likely to be male (95.8% vs. 71%, *p* < 0.001), more likely to have hypertension (89.2% vs. 78.9%, *p* < 0.001), and more likely to have been diagnosed with a stroke in the past (10.8% vs. 6.9%, *p* = 0.02). Veterans in the ALI cohort also had lower body mass index (26.7 vs. 28.3, *p* < 0.001), and were less likely to be a current or former smoker (55.9% vs. 73.4%, *p* < 0.001) compared to the COMPASS PAD cohort. There were significantly lower rates of statin therapy (78.3% vs. 83.8%, *p* = 0.024) and therapy with ACE-inhibitors or angiotensin receptor blockers (57.7% vs. 68.8%, *p* < 0.001) amongst the veterans in the ALI cohort, though there was similar utilization of antiplatelet therapy (84.6% vs. 87.7%, *p* = 0.166) between the two groups (Table 1). The initial therapy for ALI was varied, with the majority (59%) undergoing attempted revascularization. The proportion of patients undergoing endovascular (42%) and/or surgical revascularization (22%) significantly exceeded the proportion undergoing primary amputation (5%). The antiplatelet therapy based on treatment strategy was similar, though, with the majority of patients that received antiplatelet therapy after undergoing endovascular or surgical revascularization treated with clopidogrel with a smaller proportion of patients receiving the medication after primary amputation (Table 2).

### 3.2. Adverse Events

**Figure 1:**
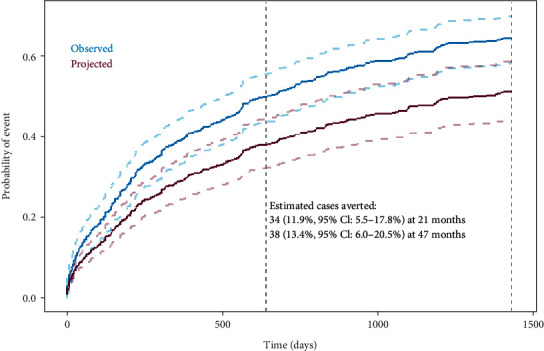
Cumulative incidence plot of the primary composite endpoint including adverse cardiovascular and limb events. (i) The projected cumulative incidence was calculated by first multiplying the observed cumulative hazard by the hazard ratio, its lower confidence interval (CI) limit, and its upper confidence interval limit reported in the COMPASS trial subgroup analysis (HR = 0.69, 95% CI: 0.56-0.85 [8]). It was then transformed to the projected cumulative incidence.

Within the observation period of 47 months, 174 veterans experienced at least one event of the composite outcome as their first event, and 27 veterans died due to noncardiovascular-related causes. Bleeding events were rare in the cohort, afflicting only 5 (1%) patients within 72 hours of hospital discharge. Using previously published data to estimate a potential benefit, 34 potential adverse events (95% CI: 15-50 cases) may have been avoided had the ALI veteran cohort been universally treated with rivaroxaban at 21 months. At 47 months, 38 potential cases (95% CI: 17-58 cases) may have been prevented. Ultimately, rivaroxaban treatment may have resulted in an 11.9% reduction in cases at 21 months (95% CI: 5.5-17.8%) and a 13.4% reduction in cases at 47 months. (95% CI: 6.0-20.5%, Figure 1).

### 3.3. Costs

The average, total cost for veterans who experienced at least one event of the composite outcome was $49,037 (95% CI: $41,479-$58,830). Thus, at 21 months, the potential cost savings of treating the ALI veteran cohort with rivaroxaban is $1.7 million (95% CI: $1.4-$2.0 million). At 47 months, the potential cost savings of rivaroxaban treatment is $1.9 million (95% CI: $1.6-$2.2 million).

## 4. Discussion

The present study evaluated the potential reduction in adverse events treated with novel anticoagulants among patients presenting with ALI. As the data demonstrate, adverse events among patients with severe PAD are high with a preponderance of cardiovascular and limb related outcomes. The application of clinical trial data to this population suggests an 11.9% reduction in these adverse events over a 21-month period if universally applied after the index presentation. Prescription of these medications after a longer follow-up period of 47 months leads to a potential 13.4% reduction in the same outcome, suggesting an even greater potential benefit for this vulnerable patient population. These data have important implications for the medical management of patients with ALI.

Though less common than other manifestations of PAD, ALI continues to represent the most devastating manifestation of this clinical entity [9, 10]. Previous studies have shown significantly higher rates of limb loss and death for patients undergoing revascularization for ALI compared to patients undergoing revascularization without a diagnosis of ALI, with a greater than twofold increase in the need for amputations and twofold increase in mortality at one year for patients diagnosed with ALI [4]. Though the scope of available treatments for ALI has expanded to include medical therapy, percutaneous endovascular treatment options, and more traditional open surgical treatment options, morbidity and mortality attributable to ALI remain a significant area or concern for peripheral vascular providers [2]. To this end, the identification of novel therapies to help combat in the fight against ALI has been of paramount importance.

Novel oral anticoagulants may provide an effective adjunct to reducing adverse events among patients with ALI. Initial investigations exploring the use of the vitamin K antagonist warfarin did not reduce adverse events in this vulnerable population [6]. However, subsequent studies have suggested that novel oral anticoagulants such as rivaroxaban may reduce the morbidity for these patients. In COMPASS, the addition of low-dose rivaroxaban (2.5 mg twice daily) in addition to aspirin therapy significantly reduced major adverse cardiovascular events in patients with known PAD or carotid disease [7]. In VOYAGER, similar benefits were again demonstrated with this dosing strategy in a similar patient set [11]. As expected, patients with ALI made up only a very small proportion of the patients included in each of these three studies, and increased instances of significant bleeding were observed in each cohort of patients receiving oral anticoagulants when compared to control groups. As such, determining a patient's bleeding risk remains an essential and important step prior to selecting anticoagulant therapy, regardless of the indication. Combining this anticoagulant therapy with potent antiplatelet agents commonly prescribed after endovascular intervention is also fraught with ambiguity. The present analysis demonstrated that a large proportion of patients with acute limb ischemia are prescribed P2Y12 inhibitors after their hospitalization, with clopidogrel the most commonly used agent. Future studies will likely assess the appropriate combination of antiplatelet therapies with novel anticoagulants to optimize outcomes for patients with peripheral artery disease, including those with acute limb ischemia.

The introduction of novel oral anticoagulants could reduce the morbidity and healthcare costs for patients with ALI. Previous data suggests that the introduction of a novel oral anticoagulant reduces the risk of subsequent adverse limb events by 1.4% in a broad population of patients with PAD [7]. Applying that reduction to a homogeneous population of patients with lower extremity, ALI could result in realization of a significant reduction in costs to the healthcare system. In the present analysis, the average cost of treating a patient who experiences at least one condition from the composite outcome is $49,037. Treatment with rivaroxaban results in marked reduction in spending, with a $1.7 million reduction in spending over 21 months and a $1.9 million reduction in spending over 47 months. Currently, the cost of rivaroxaban 2.5 mg twice daily, the dosing, which has most clearly demonstrated benefit in past trials, is $591 per month, which correlates to a cost of $12,411 dollars for 21 months and $27,777 over 47 months. At this rate, treating veterans within the ALI cohort with rivaroxaban potentially saves $1.69 million over 21 months and $1.87 million dollars over 47 months. Further investigations should be considered to evaluate whether the universal application of novel anticoagulants to this patient population realizes the calculated reduction in morbidity and healthcare costs.

### 4.1. Limitations

The present analysis should be interpreted in the context of several limitations. The cohort was initially derived from administrative billing codes to identify patients with ALI, which may not be sensitive or specific. To mitigate this limitation, the sample was then reviewed by two experienced clinicians to ensure that only patient with acute symptoms and demonstrated acute occlusion of a lower extremity artery were included in the final analysis. Unfortunately, there was significant heterogeneity in the clinical documentation for these patients such that the acute limb ischemia stage could not be consistently ascertained. The patient population in this cohort was slightly different than those in prior clinical trial investigating novel anticoagulants. In most cases, these differences suggested a higher preponderance of comorbid conditions, consistent with presentations for acute events rather than a broad population of patients with PAD. The effect of anticoagulation in reducing adverse events demonstrated in clinical trials may thus underestimate the potential benefit in this more critically ill population. It is thus possible that these results underestimate the magnitude of benefit for novel anticoagulants in patients with ALI. Finally, the data were derived from a cohort that is predominantly white males. Further studies in other populations should be considered to evaluate these findings in other healthcare systems.

## 5. Conclusions

ALI is associated with a high rate of morbidity and costs to the healthcare system. The application of novel oral anticoagulants to this patient population could result in a reduction in adverse events and costs, suggesting an opportunity for future investigation.

## Figures and Tables

**Table 1:** Baseline covariates of veterans in the ALI cohort and patients with PAD in the COMPASS trial.

	ALI cohort	COMPASS PAD cohort	*p* value
n	286	2492	
Age	66.9 (8.7)	67.9 (8.45)	0.06
Male	274 (95.8%)	1774 (71%)	<0.001
BMI	26.7 (5.4)	28.3 (5.0)	<0.001
Systolic BP	135.2 (13.6)	138.9 (18.5)	<0.001
Diastolic BP	76.1 (8.3)	77.7 (10.1)	0.003
Current or former smoker	160 (55.9%)	1829 (73.4%)	<0.001
Median cholesterol (mmol/L)	4.3 (3.5-5)	4.2 (3.6-5.0)	*N/A* ^∗^
Hypertension	255 (89.2%)	1966 (78.9%)	<0.001
Diabetes	119 (41.6%)	1100 (44.1%)	0.45
History of stroke	31 (10.8%)	171 (6.9%)	0.02
eGFR < 60 mL/min	74 (32.5%)	688 (27.6%)	0.138
*Medications*			
Antiplatelets	242 (84.6%)	2185 (87.7%)	0.166
Statin	224 (78.3%)	2088 (83.8%)	0.024
ACE-I or ARB	165 (57.7%)	1715 (68.8%)	<0.001
Beta blocker	181 (63.3%)	1477 (59.3%)	0.212
Proton pump inhibitor	146 (51%)	826 (33.1%)	<0.001
*Primary treatment strategy*			
Revascularization	168 (59%)	—	—
Endovascular	121 (42%)	—	—
Surgical	63 (22%)	—	—
Amputation	14 (5%)	—	—
None	115 (40%)	—	—

(i) Data presented as mean (standard deviation) for continuous variables or proportions for categorical variables. (ii) Abbreviations: Std Diff: absolute standardized difference. (iii) Because the full data from the PAD subgroup analysis were unavailable, we could not formally test the difference in medians between the ALI veteran cohort and the COMPASS trial PAD subgroup.

**Table 2:** Antiplatelet therapy prescribed within 90 days, stratified by primary treatment modality.

Treatment	Antiplatelet prescriptions (number)	
*Endovascular*	*Overall*	*90*
	Clopidogrel	67
	Prasugrel	1
	Ticagrelor	1
	Other	71
*Surgical*	*Overall*	*39*
	Clopidogrel	21
	Prasugrel	0
	Ticagrelor	0
	Other	33
*Amputation*	*Overall*	*9*
	Clopidogrel	5
	Prasugrel	0
	Ticagrelor	0
	Other	7
*None*	*Overall*	*74*
	Clopidogrel	34
	Prasugrel	0
	Ticagrelor	0
	Other	63

(i) Antiplatelet prescriptions are not mutually exclusive.

## Data Availability

Primary data will be available based on reasonable request but subject to the stringent privacy rules of the Department of Veterans Affairs and United States Government.

## Abbreviations

ALI: Acute limb ischemia
PAD: Peripheral artery disease
VA: Veterans Affairs.
